# Questionnaire-based computational screening of adult ADHD

**DOI:** 10.1186/s12888-022-04048-1

**Published:** 2022-06-15

**Authors:** Arthur Trognon, Manon Richard

**Affiliations:** 1 Clinicog, 185 rue Gabriel Mouilleron, Nancy, France; 2 Lorraine University, 23 Boulevard Albert Ier, Nancy, France

**Keywords:** ADHD, Diagnosis, Machine-learning, Clinical scale, Psychometrics - adult

## Abstract

**Background:**

ADHD is classically seen as a childhood disease, although it persists in one out of two cases in adults. The diagnosis is based on a long and multidisciplinary process, involving different health professionals, leading to an under-diagnosis of adult ADHD individuals. We therefore present a psychometric screening scale for the identification of adult ADHD which could be used both in clinical and experimental settings.

**Method:**

We designed the scale from the DSM-5 and administered it to *n* = 110 control individuals and *n* = 110 ADHD individuals. The number of items was reduced using multiple regression procedures. We then performed factorial analyses and a machine learning assessment of the predictive power of the scale in comparison with other clinical scales measuring common ADHD comorbidities.

**Results:**

Internal consistency coefficients were calculated satisfactorily for TRAQ10, with Cronbach’s alpha measured at .9. The 2-factor model tested was confirmed, a high correlation between the items and their belonging factor. Finally, a machine-learning analysis showed that classification algorithms could identify subjects’ group membership with high accuracy, statistically superior to the performances obtained using comorbidity scales.

**Conclusions:**

The scale showed sufficient performance for its use in clinical and experimental settings for hypothesis testing or screening purpose, although its generalizability is limited by the age and gender biases present in the data analyzed.

**Supplementary Information:**

The online version contains supplementary material available at 10.1186/s12888-022-04048-1.

## Introduction

ADHD is a multifactorial disorder presenting a heterogeneous psychopathological profile with heterogeneous neurocognitive deficits, but with core symptoms implicating function of the frontal-subcortical-cerebellar pathways that control attention, saliency, inhibitory control and response to reward [1]. This condition is classically looked as one of the most common childhood disease, but recent longitudinal studies accumulated evidences that ADHD persists into adulthood, with varying displayed characteristics among age [2–4]. In fact, while about 5% of children and adolescents are affected in the general population, this condition may persist in adulthood for at least half of these patients [5]. Several authors pointed out that this classical looking at ADHD has led to an underdiagnosis and undertreatment of adult ADHD [6]. These issues has led to strong international efforts to share information to clinician, for example in the rework of the widely-used Diagnostic and Statistical Manual of Mental Disorders - Fifth Edition [7], which highlighted the possible differential expression of ADHD throughout the patient’s lifetime, and the European consensus statement on diagnosis and treatment of adult ADHD [4], in order to facilitate the identification and the treatment of ADHD. The DSM-5 describes ADHD as an interfering, persistent disorder that manifests itself simultaneously on several levels (e.g., social, domestic or occupational). In adults in particular, it describes ADHD as extreme restlessness or intensity of activity that is exhausting to others; presenting simultaneously or not with rushed actions occurring at the moment without reflection on their possible consequences and with a risk of causing harm to the subject, with a strong propensity for reward-based functioning, and with notable difficulties in inter-rating and making long-term decisions. He emphasizes the importance of the context in the reading of the patient’s complaints and grievances, and in particular on the fact that the manifestations of the disorder must be present in more than one context, with notable variabilities according to the context of observation. However, due to the significant heterogeneity observed in adult ADHD, some authors have highlighted difficulties in establishing differential diagnoses of ADHD, especially when the question of differential diagnosis with bipolar disorder arises, where many symptoms overlap with significant comorbidity [8, 9].

Actually, the recognition of the importance of diagnosing and treating ADHD is growing, as it was recently shown that there is a higher prevalence of ADHD in some clinical populations such as addictive, forensic, and personality disorder patients [10]. In fact, in a general manner, ADHD was massively reported as a condition impairing individual interaction with his environment, increasing the risk of the occurrence of harmful events such as injuries [11], traffic accidents [12], and substance abuse [13], leading to increased healthcare utilization [2], unemployment [14], and suicide [15]. These observations pointed out the particular importance of screening within this high-risk populations.

However, ADHD is usually diagnosed through a time-consuming multidisciplinary evaluation process, including at least the visit to behavioral specialists such as psychologists and to mental functioning specialists such as neurologists or psychiatrists, increasing the diagnostic process duration which can extend over long periods of time. With the increasing need for screening, this led to the creation of psychometric auto-administered scales, such as the well-known Adult ADHD Self-Report Scale [16], which was recently updated to the DSM-5 [17], in order to shorten the diagnostic process. However, this work concerns a scale that has not been validated in French and thus cannot benefit to the French ADHD population and its need in terms of screening. Moreover, this instrument seems to have several related to its construction: firstly, the ASRS is constructed on the basis of a 5-point Likert scale, thus having a midpoint in its scaling and possibly subject to a neutrality bias, even if its existence is still debated [18, 19]; in addition, the items were constructed by adapting the terminology used in the diagnostic criteria, thus minimizing the contextual effects that may emerge during their reading by reducing them to a general presentation; and finally, the questions are presented in the form of the 2nd person plural “You”, whereas it has recently been shown that stimuli carried out using the personal pronoun “I” allow for greater immersion of the subject in relation to a situational context [20]; a measure that would seem particularly relevant in the context of ADHD and in relation to the description given in the DSM-5.

In order to meet the demands of the French ecosystems in terms of screening, we have developed an adult ADHD screening scale in 6 points built around the personal pronoun “I” and theoretically articulated around the DSM-5. Moreover, as machine-learning approaches have enabled considerable progress on a large variety of numerical problems in a wide variety of fields such as physics [13, 14], chemistry [15], and neuroscience [16, 17]; we decided to produce at the same time an algorithmic model based on the tool, in order to establish an automated screening model usable in adults, and allowing both to benefit the clinical ecosystems (i.e. the tool can be used to guide or validate the diagnostic intuition) and research (i.e. the tool can also perform an automated screening from a pre-trained model).

We thus generated 43 preliminary items based on the DSM-5 criteria (pTRAQ: Preliminary Trognon & Richard ADHD Questionnaire), and administered this scale to 110 ADHD subjects and 110 controls. We then statistically selected the most discriminating items in regard to the presence or absence of the subject’s clinical condition in order to generate the final Trognon & Richard ADHD Questionnaire French 10-items (TRAQ10). We then analyzed its psychometric properties, in terms of internal consistency, as well as factor structure, under the prism of a 2-factor model accounting for the behavioral expression of cognitive symptomatology (i.e., attention and inhibition/impulsivity). Finally, we examined the predictive power of the scale using a machine-learning approach, relying on operational metrics such as sensitivity and specificity, in comparison to other scales measuring ADHD comorbidities. We then selected three scales: the TRAQ, the target questionnaire, which was designed to evaluate behavioral traits of ADHD, as the scale we are assessing. We then selected two other scales as controls: first, the Depression Anxiety Stress Scales 21-items [21], which share dimensions with ADHD construct as it was previously shown [22–24]; then, the Scale of Adherence to the Values of the Ideal Democracy [25]; was used as a “negative-control scale”, as we thought ADHD condition should not make vary the perception of the values carried by democracy, and thus should not have link with the ADHD construct. We then performed three automated classification of ADHD diagnosis using XGBoost Classification (i.e. one algorithm for each condition), and evaluated each predicting power using classification metrics such as sensitivity, specificity, negative and positive predictive values; under the assumption that TRAQ10 questionnaire should have higher and statistically different accuracy from predictions obtained using the two others questionnaires, as this is the only one to formally measure ADHD behavioral traits.

## Material and method

### Study design

The initial questionnaire in 43-items was adapted from the DSM-5 and every item was matched to one cognitive domain affected accordingly to the behavioral expression of the symptomatology of ADHD on two domains: attention, and inhibition/impulsivity (Additional File 1). It was then computerized using the Google Form tool. Sampling was carried out randomly by distributing the questionnaire on social networks, without direct contact with the participants and on the basis of anonymous voluntary contributions. Only age, gender, socio-cultural level, diagnosis and date of completion were collected, ensuring complete anonymity for participants. The most discriminating items were selected using a stepwise backward-elimination multiple regression (Additional File 2). Then the factorial structure was tested and the norms produced (Additional File 5). Finally, three classification algorithms were trained to measure the differential performance of the screener when compared to other scales measuring or not comorbidities associated with ADHD to computationally assess discriminant validity.

### Subjects

Two hundred and twenty not preselected adults (mean age = 27.8 years, SD = 9.2; ADHD *n* = 110; controls = 110) from the general French population participated in this study through an online voluntary aleatory sampling. All questions were completed by all participants, thus, all measures were available for all subjects. The entire dataset is available in (Additional File 3).

All participants received detailed information about the study purpose and objectives, and provided online informed consent to participate in the study. All procedures were conducted in accordance with the Declaration of Helsinki and the study protocol was approved by the Institutional Review Board Commission Nationale de l’Informatique et des Libertés (registration n°2224719v0).

### Scale development: item generation and selection

Items were developed from the widely-used Diagnostic and Statistical Manual - Fifth Edition [7]. For each criterion, we generated 1 to 3 items (depending on whether an item overlapped several criteria), with the objective of bringing out a personal representation on which the participant could make a judgment on the concordance or otherwise between the induced representation and the subject’s subjective feelings. 43 items were thus generated for the preliminary version of the scale. We selected a 6-points Osgood-style scale, ranging from 1 “Don’t look like me at all.” to 6 “Looks just like me.”, in order to avoid neutrality bias, even if its existence is still currently debated [18, 19].

We then conducted a reiterative stepwise top-down regression multiple linear regression (diagnosis~item_i_) in order to elucidate the factors by which the dependent variable was about to be influenced and for selecting discriminating items to highlight the diagnosis of ADHD.

### Internal consistency and reliability

Internal consistency and reliability of the remaining items was examined by Cronbach’s alpha. Reasonable acceptability criterion was set to .70 ≤ ɑ ≤ .90 with exceeding lower bound meaning a low reliability, and exceeding higher bound meaning too many similar items, decreasing the scale’s true reliability [26, 27].

### Factor structure

In order to test our 2-factors model for TRAQ10 and assess construct validity, we conducted a confirmatory factor analysis. Generalized least squares method was performed in order to test the fit capability of the factor structure. Model fit was assessed using the following fit indices: we used the χ2 test statistic for absolute fit; the comparative fit index (CFI) and Tucker-Lewis Index (TLI) for fit relative to a null model [28, 29]; the Standardized Root Mean Square Residual (SRMR [30]) and the Root Mean Square Error of Approximation (RMSEA [31]) for overall fit. Accordingly to Hu & Bentler (1999) [32], we assumed that our 2-factors model fit well if CFI > .95; TLI > .95; RMSEA<.06 and SRMR<.08. All statistical analyses were coded in R with Lavaan library and interpreted in RStudio v1.0.143.

### Evaluation of the scale’s discriminating power

To evaluate the scale’s ability to capture the ADHD diagnosis, we chose to base our analysis on a machine-learning model. Machine-learning approach consists in training an algorithm on a subset of the whole dataset and testing the algorithm on an independent subset. Here, we used a XGBoost classification algorithm to assess the predictive power of the TRAQ10 participant’s responses in order to diagnose and exclude ADHD diagnosis in comparison to the other scales used in this research. XGBoost is a scalable end-to-end tree boosting system which is aware about sparsity and weight quantile sketch for approximate tree learning [33]. In this study, we used grid search techniques to set the hyperparameters of all the classification algorithms, and we performed a k-Fold (with k = 10) cross validation in order to validate the model. Machine-learning algorithms were coded in Python and statistical analysis were coded in R and interpreted in RStudio v1.0.143.

After data collection, the whole dataset was split randomly to constitute an independent training set (*n* = 154) and test set (*n* = 66) using Sci-Kit Learn Libraries with a 70/30 ratio. For each scale (i.e. TRAQ10, DASS21, and AVDI), a XGBoost algorithm classifier was constructed based on training set data. A grid search was performed in order to optimize each classifier. We optimized the following hyperparameters: the eta (from 0.1 to 1 by step of .05); the gamma (from 0.1 to 1 by step of .005); the max depth (from 1 to 10 by step of 1); the min child weight (from 0 to 1 by step of .25), the subsample (from 0.1 to 1 by step of .01), the sampling method (two levels: uniform or gradient based), the alpha (from 0 to 1 by step of .25), the lambda (from 0 to 1 by step of .25), the refresh leaf (two level: 0 or 1), and the colsample by tree, level, and node (each from 0.1 to 1 by step of .0025). The scoring method was set for accuracy optimization. Hyperparameters settings are shown in Additional File 4. Then, a 10-fold cross-validation was performed for each algorithm on the remaining test set in order to evaluate models’ performances in terms of accuracy. Sensitivity and specificity values were obtained for each algorithm. The accuracy values obtained for each algorithm were then statistically compared by two-ways ANOVA test, with prediction’s source (three levels: TRAQ10; DASS21; or AVDI) and accuracy metric as parameters in order to verify whether the algorithm based on the hyperactivity scale showed significantly better performance than those based on the other two scales (i.e. DASS and AVDI). Random state was set to 0 in all conditions.

## Results

### Descriptive statistics

Descriptive statistics of the study sample are shown in Table 1 and full norms for each population expressed in centiles are available in Additional File 5. Results showed that ADHD patients reported higher scores compared to controls for all dimensions.

**Table 1:** Descriptive statistics of the study sample

Parameter	ADHD (*n* = 110)	Controls (*n* = 110)	*p-value*
Age	34.09 (±9.35)	21.6 (±2.42)	***<.001***
Men	19	19	–
Women	91	91	–
Socio-cultural level	2.56 (±1.05)	2.92 (±.68)	***.002***
Attention	23.13 (±4.26)	15.09 (±5.5)	***<.001***
Impulsivity	23.7 (±3.78)	13.05 (±5.01)	***<.001***
Full scale	46.87 (±7.13)	28.6 (±9.82)	***<.001***

### Multiple linear regression

Multiple linear regression analysis (stepwise elimination procedure) using diagnosis as the dependent variable and patients’ scores on pTRAQ (project questionnaire) as independent variables (diagnosis~item_i_) revealed that ADHD makes significatively vary scores on pTRAQ_3_ [*β* = .047, *p* = .02], pTRAQ_11_ [*β* = .065, *p* = .007], pTRAQ_41_ [*β* = .034, *p* = .03], pTRAQ_4_ [*β* = −.06, *p* = .02], pTRAQ_1_ [*β* = −.05, *p* = .001], pTRAQ_28_ [*β* = −.08, *p* < .001], pTRAQ_26_ [*β* = −.003, *p* = .01], pTRAQ_24_ [*β* = .005, *p* = .008], pTRAQ_14_ [*β* = −.10, *p* < .001], and pTRAQ_7_ [*β* = .003, *p* = .02]. Measures obtained on the 10 remaining items out of the 43 was [*F*_*(10,209)*_ = 38.8, *p* < .001, R^2^ = .6499 and R^2^_adjusted_ = .6331].

### Internal consistency and reliability

Results concerning internal consistency and reliability are presented in Table 2. Data shown than the TRAQ10 questionnaire carry high internal consistency and reliability even when an item is dropped.

**Table 2:** Internal consistency and reliability of the TRAQ10 if an item is dropped

Project ID	Screener ID	Reliability if dropped
pTRAQ3	TRAQ1	.89
pTRAQ11	TRAQ2	.89
pTRAQ41	TRAQ3	.90
pTRAQ4	TRAQ4	.88
pTRAQ1	TRAQ5	.91
pTRAQ28	TRAQ6	.90
pTRAQ26	TRAQ7	.90
pTRAQ24	TRAQ8	.89
pTRAQ14	TRAQ9	.89
pTRAQ7	TRAQ10	.90

The Cronbach’s alpha was measured at .9 [CI_95%_ = .89–.92] for the full TRAQ10 questionnaire. When each of the TRAQ10 items was removed from the analysis in order to assess robustness, Cronbach’s alpha remained high (varying from .89 to .90 with mean_ɑ_ = .89, SD = .008). All measures were above the minimum acceptable rate of .70 and was close from the maximum expected value of .9.

### Confirmatory factor analysis

Confirmatory factor analysis suggested a good model fit with the TRAQ10 questionnaire. Significant items selected by the multiple regression were regrouped in two factors for the two behavioral modules described in the DSM-5. Items TRAQ1;2;4;8;10 was combined in the “Attention” factor, and items TRAQ3;5;6;7;9 were combined in the “Inhibition/Impulsivity” factor. Confirmatory factor analysis suggested that the 2-factors model has an acceptable fit with the TRAQ10 questionnaire, with metrics that remains slightly below the pre-defined cut-off [*χ2*_*(34)*_ = 138.45, *p* < .001, CFI = .91, TLI = .88, RMSEA = .11, SRMR = .058].

Table 3 shown standardized factor loadings for the TRAQ10. Analysis revealed than all of the standardized factor loading ranged from .7 to .89 for attention, and from .49 to .83 for impulsivity module. Correlation between attention and impulsivity was significant and measured at *r* = .98.

**Table 3:** Standardized factor loadings of the 2-factor model of the TRAQ10

Project ID	Screener ID	Attention	Inhibition/Impulsivity
pTRAQ3	TRAQ1	.75	–
pTRAQ11	TRAQ2	.86	–
pTRAQ41	TRAQ4	.89	–
pTRAQ4	TRAQ8	.74	–
pTRAQ1	TRAQ10	.7	–
pTRAQ28	TRAQ3	–	.58
pTRAQ26	TRAQ5	–	.49
pTRAQ24	TRAQ6	–	.62
pTRAQ14	TRAQ7	–	.5
pTRAQ7	TRAQ9	–	.83

Given the notable imbalance between the number of men and women who participated in the study, we also conducted the analysis only in the female sample. This analysis is available in Additional File 6.

### Predictive power of the questionnaire and discriminant validity

**Fig. 1:**
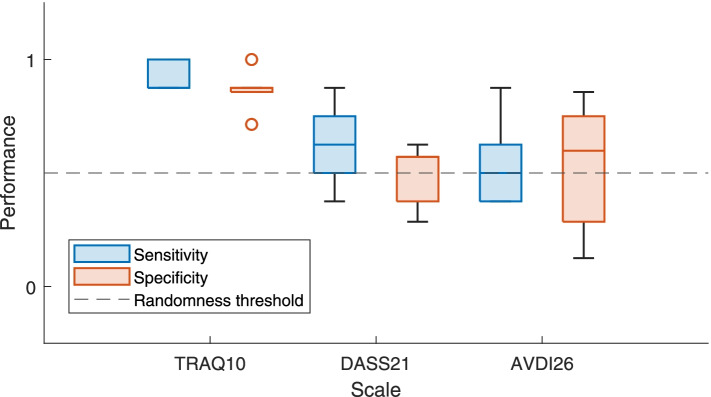
Results of the detection metrics in 10-fold cross-validation

Results for the 10-fold cross-validation of the classification task are presented in Fig. 1 and Table 4. Data showed that the algorithm was able to identify ADHD diagnosis in patients and exclude it in controls based only on the participants’ responses to the TRAQ questionnaires and with high accuracy.

**Table 4:** Tukey post-hoc analysis results for classification performances

Scale	TRAQ10	DASS21	AVDI26
TRAQ10	–	***p*** **< .001**	***p*** **< .001**
DASS21	***p*** **< .001**	–	*p* = .39
AVDI26	***p*** **< .001**	*p* = .39	–

The XGBoost classifier which was trained on the TRAQ10 data of 154 out of the 220 randomly selected participants was able to identify group appurtenance of the remaining participants with an accuracy of .98, identifying correctly 36 of the 36 ADHD participants and 29 of the 30 controls [sensitivity = .97; specificity = 1; PPV = 1; NPV = .97]. These results differ from whose obtained in other conditions: first, for DASS21 predictions, the classifier was able to identify correctly the group appurtenance of remaining participants with an accuracy of .74, identifying correctly 27 of the 36 ADHD participants and 22 of the 30 controls [sensitivity = .77; specificity = .71; PPV = .75; NPV = .73]. Finally, for AVDI predictions (negative control), the classifier was able to identify the group appurtenance of the remaining participants with an accuracy of .59, identifying correctly 23 of the 36 ADHD participants and 16 of the 30 controls [sensitivity = .62; specificity = .55; PPV = .64; NPV = .53].

Further two-ways ANOVA on 10-fold cross validation accuracy measures (accuracies~source) revealed a significant effect for source [F [*2,27*]=29.81,*p* < .001], and further Tukey’s post-hoc analysis revealed significant differences between predictions from TRAQ10 and DASS21 [*p* < .001] and from TRAQ10 and AVDI [*p* < .001], but not between predictions from DASS21 and AVDI [*p* = .39].

These results suggest that participant’s responses to TRAQ10 questionnaire are sufficient to predict ADHD diagnosis with high accuracy. In fact, even if other questionnaires (i.e. DASS21 and AVDI) are able to perform beyond chance, both showed similar lower and statistically different accuracies when compared to the TRAQ10 performances.

## Discussion

The present study verified the reliability and the construct validity of the Trognon & Richard ADHD Questionnaire 10-items French version for Adults (TRAQ10) and allowed the production of a tool that can be used both in clinical routine and in experimental settings.

Cronbach’s alpha values suggested that the TRAQ10 was internally reliable, with measured alpha remaining in the .70 ≤ ɑ ≤ .90 intervals recommended by (Bland & Altman, 1997; DeVellis, 2003) [26, 27], except when the item 5 is dropped, and where the measured alpha exceeds the upper limit of .01.

Further confirmatory factor analysis supported our two-factors structure for the TRAQ10 questionnaire, with items TRAQ1;2;4;8;10 grouped in the “Attention” factor, and TRAQ3;5;6;7;9 grouped in the “Inhibition/Impulsivity” factor. Analysis showed that this model has an acceptable fit according to the standards defined by (Hu & Bentler, 1999) [32]. Furthermore, all items highly loaded on their attributed factors, and all factors correlated with the others. However, these factorial measures had to be replicated in neuropsychological studies and evaluated for their relatedness to cognitive impairments.

In our analysis, we used machine-learning procedures to analyze the pattern of participant’s responses to the TRAQ10 questionnaire among ADHD and neurotypical population, and compared these results with two others scales to validate formally its predictive power. The XGBoost machine-learning classifier trained on the data of the TRAQ10 questionnaire performed with high accuracy and very few rates of false positive. This model showed better (and statistically different) performances from those based on the two others scales (i.e anxiety and democracy adhesion) scales, demonstrating its predicting power in order to label ADHD attribute in comparison with other scales which carry (i.e. DASS21) or not (i.e. AVDI) direct or indirect link with ADHD construct; thus outlining the manner in which this type of classifier could facilitate the professional’s diagnostic process of ADHD, given its automated design, allowing it to be used during a consultation without the need to manually calculate results or use statistical tables, which is a significant gain of time, less susceptible to human error, and directly implementable. In addition, its automated design would pave the way for its use in a dynamic, mobile, fast, and flexible manner, particularly in the research ecosystems which could use this type of computational screener to automatically assign the clinical label according to a pre-trained model, making it possible to carry out large-scale psychometric studies, without prior knowledge of the population to be sampled. However, future work on this classifier is still needed, in particular on its differential performance when applied to datasets carrying several and/or confounding clinical conditions (such as bipolar disorder or Asperger’s syndrome).

More generally, we believe that these computational screening strategies will have an important role to play in the current context of Evidence-Based Practices, especially in the context of treatment, which is one of the major themes discussed about ADHD in these fields of expertise [34]. Indeed, in the same way that computational techniques have been used to predict the optimal parameters of deep-brain stimulation and functional imaging [35], one could imagine predicting the possible benefits of different interventions (e.g. pharmacology VS psychotherapy VS combined), based on the patient’s response pattern, under the assumption that the patient’s response pattern should be conditioned by his or her cognitive state. Similarly, one could imagine combined uses of different tools trained on different clinical conditions to calculate the probabilities of one or several given clinical conditions taken simultaneously.

Nevertheless, the present study suffers from several limitations that reduce its generalizability. First, a strong age bias (statistically significant) was measured simultaneously with a weak socio-cultural level bias (statistically significant) which may have biased the training of the algorithms. We were also unable to obtain the same number of individuals for each sex, despite having obtained the same number of individuals of each sex for each group, leading us to consider an established validity for women, while remaining partial for men. We did not measure ethnicity as well. Furthermore, we did not perform a positive control or convergent validity experiment by measuring the performance of tools already available in French or English that could have been translated and we were not able to check for corrupted data, given the computerized aspect of the administration of the questionnaires, thus not allowing us to check for the presence of artifactual or erroneous data. Finally, we did not cross-reference the typical symptoms mentioned by the DSM-5 with other classifications, such as those proposed by Barkley or Fedele and their respective collaborators [14, 36], and could have helped to outline ADHD symptoms not covered by the DSM-5 (or being particularly appropriate in the context of adult ADHD, in contrast to the presentation that this condition may have during childhood).

## Conclusion

We concluded from these analyses that the TRAQ10 can be used to attribute ADHD attribute in a French survey sample. From a clinical point of view, we believe that this scale could be used during a first consultation or at the end of the consultation to support the health professional’s clinical expertise and serve as a decision aid in deciding whether to pursue a multidisciplinary diagnostic process. In contrast, from an experimental point of view, we believe that this tool could be used to automatically label the attribute ADHD in a given sample, and thus allow to perform psychometric without a priori knowledge of the sampled population, but whose generalizability will be limited by the biases present in the study sample.

## Supplementary Information

**Additional file 1.**

**Additional file 2.**

**Additional file 3.**

**Additional file 4.**

**Additional file 5.**

**Additional file 6.**

## Data Availability

The dataset used during the study is available in (Supplementary File 1).
