# Supplementary material for: Questionnaire-based computational screening of adult ADHD
Source: BMC Psychiatry. 2022 Jun 15;22:401. doi: 10.1186/s12888-022-04048-1 (PMC9202159; doi:10.1186/s12888-022-04048-1)
Supplement: Supplementary file 4 — Additional file 4. [file 12888_2022_4048_MOESM4_ESM.docx]

**Questionnaire-based computational screening of adult ADHD.**

**Arthur Trognon* (1, 2) & Manon Richard* (1, 2)**

**Additional File 4**

| **Parameter** | **TRAQ10** | **DASS21** | **AVDI26** |
| --- | --- | --- | --- |
| Eta | .95 | .9 | .75 |
| gamma | .095 | .4 | .75 |
| max_depth | 3 | 3 | 4 |
| min_child_weight | .9 | 1 | 1 |
| max_delta_step | 1 | 1 | 1 |
| subsample | .49 | 1 | 1 |
| sampling_method | Uniform | Uniform | Uniform |
| alpha | 0 | 0 | 0 |
| lambda | 0 | 0 | 0 |
| refresh_leaf | 0 | 0 | 0 |
| colsample_bytree | .35 | .35 | .35 |
| colsample_bylevel | .0325 | .0325 | .0325 |
| colsample_bynode | .225 | .175 | .15 |

Supplementary table 1 : Grid search hyperparameter tuning for each algorithm
