# Supplementary material for: Questionnaire-based computational screening of adult ADHD
Source: BMC Psychiatry. 2022 Jun 15;22:401. doi: 10.1186/s12888-022-04048-1 (PMC9202159; doi:10.1186/s12888-022-04048-1)
Supplement: Supplementary file 5 — Additional file 5. [file 12888_2022_4048_MOESM5_ESM.docx]

**Questionnaire-based computational screening of adult ADHD.**

**Arthur Trognon* (1, 2) & Manon Richard* (1, 2)**

**Additional File 5**

| Centile (Attention) | ADHD | Controls |
| --- | --- | --- |
| 1 | 13.09 | 6 |
| 5 | 15 | 7 |
| 10 | 17 | 8 |
| 15 | 19 | 9.35 |
| 20 | 20 | 11 |
| 25 | 20.25 | 11 |
| 30 | 21.7 | 11.7 |
| 35 | 22 | 12 |
| 40 | 22 | 12 |
| 45 | 23 | 14 |
| 50 | 23.5 | 14.5 |
| 55 | 24 | 16 |
| 60 | 25 | 16.4 |
| 65 | 25 | 17 |
| 70 | 25.3 | 18 |
| 75 | 26 | 19 |
| 80 | 27 | 19 |
| 85 | 27.65 | 20.65 |
| 90 | 28.1 | 23 |
| 95 | 30 | 25 |
| 99 | 30 | 27.89 |

Supplementary Table 2 : Normative data for the TRAQ10 Attention subscale expressed in centiles.

| Centile (Impulsivity) | ADHD | Controls |
| --- | --- | --- |
| 1 | 14.09 | 6 |
| 5 | 18 | 7 |
| 10 | 19 | 8 |
| 15 | 20 | 8 |
| 20 | 21 | 8.8 |
| 25 | 21 | 9 |
| 30 | 22 | 10 |
| 35 | 22 | 11 |
| 40 | 22.6 | 12 |
| 45 | 24 | 13 |
| 50 | 24 | 13 |
| 55 | 24 | 13.95 |
| 60 | 25 | 14 |
| 65 | 26 | 15 |
| 70 | 26 | 16 |
| 75 | 26.75 | 17 |
| 80 | 27 | 18 |
| 85 | 27.65 | 19 |
| 90 | 28 | 20.1 |
| 95 | 29.55 | 23.1 |
| 99 | 30 | 25.91 |

Supplementary Table 3 : Normative data for the TRAQ10 Impulsivity subscale expressed in centiles.

| Centile (Full scale) | ADHD | Controls |
| --- | --- | --- |
| 1 | 30.09 | 12.09 |
| 5 | 35 | 15 |
| 10 | 36 | 16 |
| 15 | 41 | 19 |
| 20 | 42 | 19 |
| 25 | 42 | 20 |
| 30 | 43 | 22 |
| 35 | 44 | 24.15 |
| 40 | 45 | 26 |
| 45 | 46 | 27 |
| 50 | 47 | 28 |
| 55 | 48 | 29 |
| 60 | 49 | 29.4 |
| 65 | 50 | 31.85 |
| 70 | 51.3 | 34 |
| 75 | 52 | 35.75 |
| 80 | 52 | 37 |
| 85 | 54 | 39 |
| 90 | 56 | 42.1 |
| 95 | 59 | 45.55 |
| 99 | 60 | 51.91 |

Supplementary Table 4 : Normative data for the full TRAQ10 questionnaire expressed in centiles.
