# Supplementary material for: Questionnaire-based computational screening of adult ADHD
Source: BMC Psychiatry. 2022 Jun 15;22:401. doi: 10.1186/s12888-022-04048-1 (PMC9202159; doi:10.1186/s12888-022-04048-1)
Supplement: Supplementary file 6 — Additional file 6. [file 12888_2022_4048_MOESM6_ESM.docx]

**Questionnaire-based computational screening of adult ADHD.**

**Arthur Trognon* (1, 2) & Manon Richard* (1, 2)**

**Additional File 6**

| **Item** | **Reliability if dropped** | **Attention** | **Inhibition/Impulsivity** |
| --- | --- | --- | --- |
| TRAQ1 | .9 | .71 | - |
| TRAQ2 | .9 | .71 | - |
| TRAQ4 | .9 | .77 | - |
| TRAQ8 | .9 | .78 | - |
| TRAQ10 | .91 | .67 | - |
| TRAQ3 | .9 | - | .77 |
| TRAQ5 | .9 | - | .62 |
| TRAQ6 | .9 | - | .73 |
| TRAQ7 | .9 | - | .77 |
| TRAQ9 | .9 | - | .73 |

Table 1: Internal consistency, reliability, and standardized factor loadings of the 2-factor model in women. Confirmatory factor analysis suggested that the 2-factors model has an acceptable fit with the TRAQ10 questionnaire, with metrics that remains slightly below the pre-defined cut-off [χ2_(34)_=254.53, p<.001, CFI=.79, TLI=.72, RMSEA=.19, SRMR=.08].
